# Toward Bimetallic Nanowire Arrays with Controlled Compositions Using Block Copolymer Films: The Interplay Between Metal Precursors

**DOI:** 10.1002/anie.202512695

**Published:** 2025-08-29

**Authors:** Ofer Burg, Carmel Cohen, Roy Shenhar

**Affiliations:** ^1^ The Institute of Chemistry and the Harvey M. Krueger Family Center for Nanoscience and Nanotechnology The Hebrew University of Jerusalem Jerusalem 9190401 Israel

**Keywords:** Bimetallic structures, Block copolymers, Liquid phase infiltration, Nanowires, Thin films

## Abstract

Metallic nanowire (NW) arrays are promising components for nanotechnology owing to their elongated, continuous, and directional structure. *Bimetallic* NWs present synergistic effects that are advantageous for applications in catalysis and magnetism. Yet, creating bimetallic NW *arrays* requires concomitant control over composition and organization. Block copolymer films, which exhibit periodic arrays of nano‐domains, provide templates that satisfy this requirement. Impregnating the film using two metal precursors followed by plasma treatment enabled us to prepare NW arrays with different bimetallic compositions. The NW composition was found to depend on the composition of the impregnation solution in a nontrivial way, which was strongly influenced by the type of metals used. Studying the behavior of three model metal pairs unraveled different characteristic co‐impregnation behaviors, which we could associate with metal‐ligand and metal‐metal interactions. The insights gained in this study allow us to create bimetallic NW arrays with compositions tailored to the desired properties.

## Graphical abstract

Co‐impregnation of block copolymer films enables the creation of ordered arrays of bimetallic nanowires (NWs). The dependence of the NW composition on the feed revealed direct and indirect modes of mutual influence between the metal precursors stemming from the types of interactions involved. The insights gained now enable to design and fabricate arrays of multimetallic nanostructures with both complex morphologies and controlled compositions.

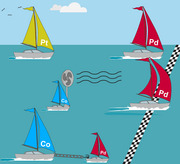

## Introduction

Metallic nanowires (NW) represent a unique type of nanotechnological building blocks. Unlike their nanoparticle counterparts, they are confined to nanoscale dimensions only in two dimensions, whereas the third dimension is extended and usually spans microns and even millimeters in length.^[^ ^1^ ^]^ This characteristic enables them to connect the functionality derived from nanoscale confinement (e.g., unique electronic and optical properties, high surface‐to‐volume ratio) to the macroscopic world and thus exploit their non‐nanoscopic properties (e.g., conductivity and plasmon propagation).^[^ ^2^, ^3^, ^4^, ^5^ ^]^

Bimetallic NWs represent a specifically intriguing class of NWs because the properties of bimetals often differ from a simple combination of the properties of their constituents. In general, the synergistic effects between the two metal components in alloys offer improved performance for various applications. For example, the magnetic properties of Ni─Fe alloys vary greatly with composition, showing a drastic increase in susceptibility and permeability around certain Ni fractions.^[^ ^6^, ^7^ ^]^ Similarly, the oxygen reduction activity of Pt─Co nanoparticles increases at around 1:1 ratio between the metals.^[^ ^8^, ^9^ ^]^ Many additional bimetallic pairs are known in which certain compositions show improved catalytic, magnetic, plasmonic, or electrochemical properties.^[^ ^9^, ^10^, ^11^, ^12^, ^13^, ^14^, ^15^, ^16^, ^17^, ^18^ ^]^ Various methods are known for creating bimetallic *nano*‐structures, such as co‐assembly of nanoparticles,^[^ ^19^, ^20^, ^21^ ^]^ solution synthesis,^[^ ^22^, ^23^ ^]^ atomic layer deposition,^[^ ^24^, ^25^ ^]^ and galvanic displacement.^[^ ^26^ ^]^ Yet, creating arrays of bimetallic NWs has been rarely explored so far.

The ability to prepare NWs in organized arrays on a two‐dimensional surface rather than being randomly scattered on it is important for applications such as sensors and electrochemical devices^[^ ^27^ ^]^ because it offers the opportunity to exploit the entire surface area of the NWs and increase cost‐effectiveness. One promising approach for fabricating organized NW arrays relies on block copolymer films as templates. Block copolymers consist of sequences of chemically distinct comonomers, which microphase separate and give rise to periodic nanostructures with typical periodicities of a few tens of nanometers. The morphology of the block copolymer is dictated mainly by the relative lengths of the blocks, and the functionality of each block is set by the choice of monomer types and their reactivity.^[^ ^28^ ^]^ Asymmetric block copolymers often feature cylinders of the minority component embedded in the matrix of the majority component. Casting a solution of asymmetric block copolymer allows to orient the cylindrical domains either parallel or perpendicular to the substrate, depending on the film thickness and the conditions used for annealing.^[^ ^29^ ^]^

Buriak et al. have pioneered the utility of block copolymer films as templates for the creation of metal NWs.^[^ ^30^, ^31^ ^]^ In their approach, a thin film of polystyrene‐*block*‐poly(2‐vinylpyridine) (PS‐*b*‐P2VP) featuring P2VP as the minority component is immersed in a solution containing negatively charged complexes of the metal precursors under acidic conditions. The protonated pyridinium groups in the film attract the negatively charged metal complexes, and thus the P2VP domains become impregnated with the metal precursors. A subsequent plasma step removes the organic matter. The metal precursors, acting as electron sinks, get reduced in the process to form metallic superstructures organized in an array that corresponds to the morphology and domain orientation of the impregnated P2VP domains in the film before plasma. When the film consists of P2VP cylinders lying parallel to the substrate, the process yields an array of periodically spaced NWs. Building on Buriak's foundational approach, elegant procedures using multistep fabrication schemes were devised to create increasingly sophisticated multicomponent NW arrays,^[^ ^32^ ^]^ mesh structures,^[^ ^33^, ^34^, ^35^, ^36^ ^]^ and segmented bimetallic NWs.^[^ ^37^ ^]^ The modularity of this approach also enabled the fabrication of hybrid nanoparticle‐NW structures.^[^ ^38^, ^39^ ^]^

We have recently shown that the sequential introduction of two metal precursors to a PS‐*b*‐P2VP film consisting of P2VP cylinders oriented parallel to the substrate, followed by plasma treatment, resulted in NW arrays that featured alternating NW compositions, where every second NW was bimetallic.^[^ ^39^ ^]^ The composition of the bimetallic NWs was set by the conditions and limitations of the assembly process, and raised questions about the interrelationship between the metal precursors during the second impregnation process and its influence on the resulting NW composition. Previous reports have shown that the simultaneous introduction of two metal precursors to the polymer film leads to bimetallic nanostructures.^[^ ^40^, ^41^ ^]^ Yet, the underlying principles that dictate the bimetallic composition are not yet understood. Given the potential of bimetallic NWs for nanotechnological applications and the crucial role of their composition on their properties, we set out to explore the process of co‐impregnation (namely, the simultaneous impregnation by immersing the polymer film in a solution containing both metal precursors) and its ability to obtain different compositions in a controlled manner. The results unraveled a non‐trivial, rather intricate behavior.

## Results and Discussion

**Figure 1:**
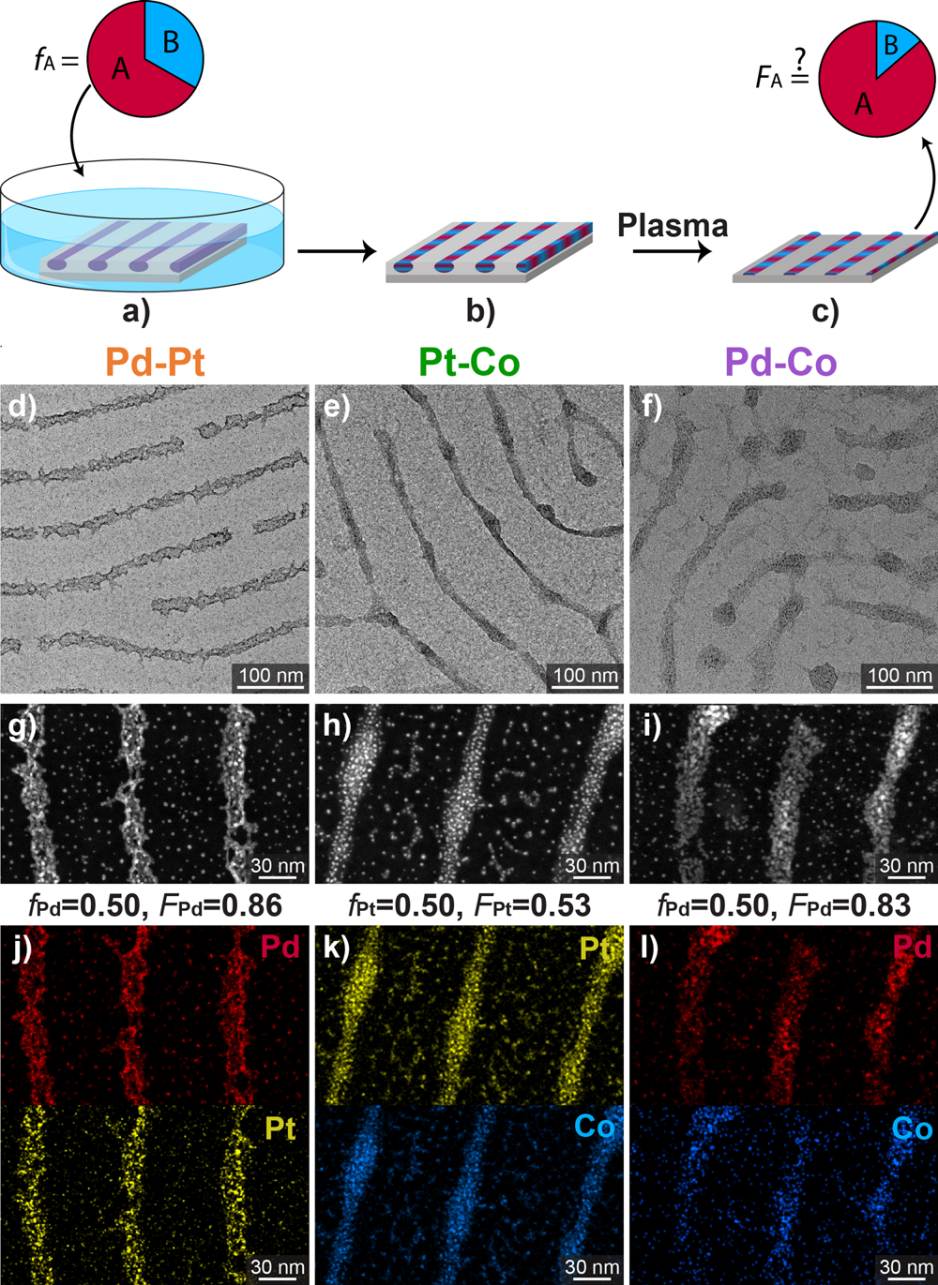
a)–c) Illustration of the co‐impregnation process for fabricating bimetallic nanowire arrays: a) The PS‐*b*‐P2VP film is immersed in a solution containing both metal precursors with a given composition *f*_A_. b) The polymer film is washed and dried, with the metal precursors absorbed selectively in the P2VP phase. c) Following plasma treatment, the composition *F*_A_ of the resulting bimetallic NWs is determined. HR‐TEM images d)–f), STEM images g)–i), and EDX elemental maps j)–l) of bimetallic NW arrays made using block copolymer templates. The metal fractions in the impregnation solution and in the resulting nanowires are denoted above the elemental maps.

Figure 1a–c describes the co‐impregnation experiment, where solutions containing two metal precursors at different ratios were used to impregnate films of microphase‐separated PS‐*b*‐P2VP, leading to periodic arrays of bimetallic NWs after plasma treatment. Keeping the total concentration of metal precursors constant while varying the composition *f*_A_ of the impregnation solution resulted in arrays of NWs with different compositions *F*_A_ (both quantified by the mole fractions of metal A), which was determined using energy dispersive X‐ray spectroscopy (EDX).

In this work, we focused on three model metal precursors: Na_2_PdCl_4_, K_2_PtCl_4_, and K_3_Co(CN)_6_. These metal precursors are stable and do not form a precipitate when mixed in solution. Literature data shows that all three pairs of the corresponding metals form miscible solids (i.e., solid solutions) at equilibrium,^[^ ^42^ ^]^ and their alloys are commonly used for catalysis.^[^ ^8^, ^9^, ^11^, ^12^, ^14^, ^15^ ^]^ The aspect of magnetism provides an additional motivation for studying cobalt‐containing bimetals.

Thin films of PS‐*b*‐P2VP (*M*_n_ 188 kDa, PDI 1.18, 71.8 %wt PS) were cast from chloroform solution on silicon substrates to create ∼30 nm‐thick films. The films were annealed under saturated chloroform vapor, giving rise to a monolayer of P2VP cylinders lying parallel to the substrate. The films were then co‐impregnated for 24 h with solutions of Pd─Pt, Pt─Co, or Pd─Co precursor pairs, varying the fraction of each metal in the solution but keeping the total precursor concentration constant at 2 mM. Figure 1d–f shows high‐resolution transmission electron microscopy (HR‐TEM) images of the bimetallic NW arrays formed after the plasma treatment.^[^ ^43^ ^]^ Elemental mapping of the NWs (Figure 1j–l) confirmed that they are bimetallic, comprising the metals that were used in the impregnation solution (termed “feed” hereafter). For clarity, each metal is indicated throughout the manuscript with a primary color (red, yellow, and blue for Pd, Pt, and Co, respectively), and each bimetallic system is indicated by the secondary colors corresponding to the primary colors used to denote its respective components (i.e., orange, green, and purple for Pd─Pt, Pt─Co, and Pd─Co, respectively).

All the NW samples in Figure 1 were made with solutions containing 1 mM of each of their corresponding metals (*f* = 0.50). Yet, the resulting NWs featured a completely different composition for each metal pair, ranging from about 50% of each metal in Pt─Co to ∼85% Pd in Pd─Pt. This points to a large difference in the way each metal precursor interacts with the block copolymer film during the impregnation process. It should be noted that the difference in the resulting NW composition did not directly reflect the different absolute concentration of each metal precursor. Measurements performed on polymer films impregnated for 24 h with each metal precursor separately over the concentration range of 0.25–20 mM showed similar content of absorbed metal within experimental error (not shown). This confirms that the determined *F* values represent saturation conditions, where the overall composition of absorbed metal precursors depends only on the fractions of metal precusros in the impregnation solution and not on the absolute concentration of each precursor.

Quantitative analysis of the NW composition was difficult to obtain due to substrate artifacts in the EDX spectra that lowered the accuracy of the measurement. The NW arrays were not mechanically stable to enable their analysis without a substrate. The block copolymer films, on the other hand, were proven robust enough to be transferred to an uncoated TEM grid and remained intact even in the absence of an underlying substrate. This enabled the EDX analysis of the metal composition in the polymer films without the intervention of substrate artifacts (see discussion and Figure S1). We have found that the bimetallic composition in the polymer film before and after plasma treatment remained the same within experimental error (see Figure S2), and therefore applies to the composition of the NWs. Hence, all the EDX‐determined composition data mentioned hereafter reflect the compositions of the impregnated polymer films.

**Figure 2:**
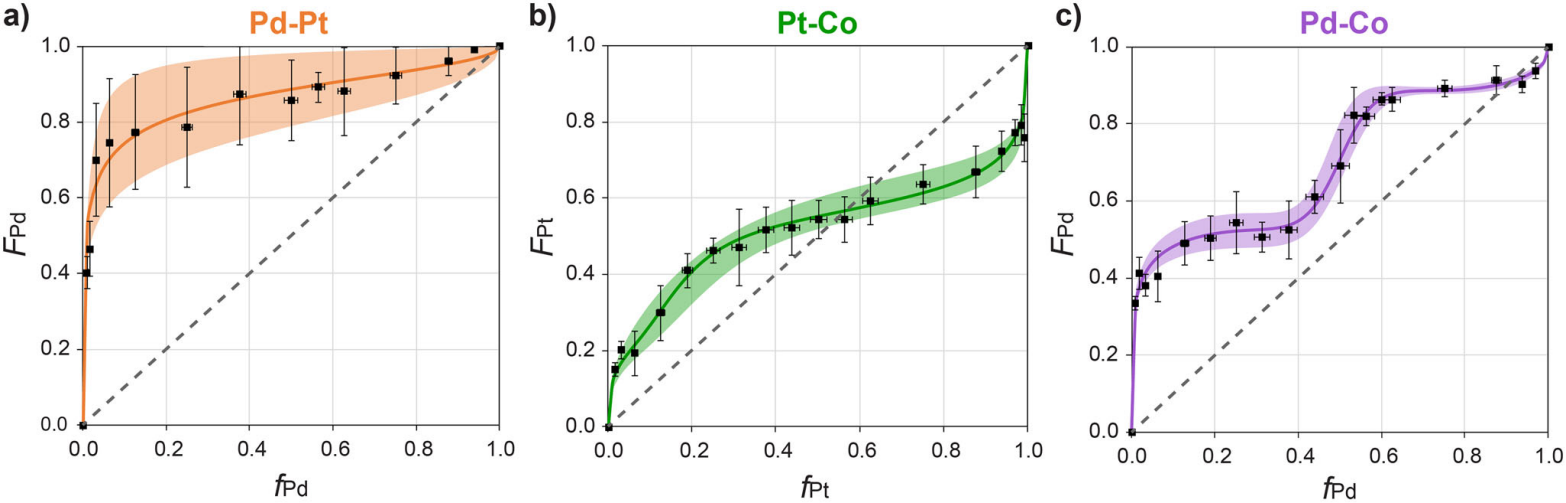
Dependence of the bimetallic composition inside the block copolymer film on the composition of the impregnation solution (feed) for each metal pair: a) Pd─Pt; b) Pt─Co; c) Pd─Co. The endpoints in each graph correspond to impregnation with a single metal. The solid lines represent the RMS fitting of the data points to Equation 1 (see below), with shaded areas marking the fitting uncertainty. Error bars denote the standard deviation from 3 to 11 sample repetitions. A dashed line representing *f* = *F* (i.e., a composition that is equal to the feed) is shown for reference.

Figure 2 shows the dependence of the resulting metal composition in the film on the composition of the impregnation solution. Each data point represents an average of 3–6 locations in each film and 3–11 sample repetitions. The curves highlight three fundamentally distinct behaviors.

The curve for Pd─Pt (Figure 2a) is asymmetrically shaped, featuring a majority of absorbed Pd for most feed compositions. The scatter in the *F*_Pd_ values determined for each feed composition is relatively large, especially for Pd‐poor feed solutions (i.e., low *f*_Pd_). It should be noted that all the individual Pd─Pt impregnation series, for which the average *F*_Pd_ values and standard deviations were calculated, followed the same trend with little variation within each sample (see Figure S3a). This suggests that whereas each sample was compositionally homogeneous, the impregnation of the Pd─Pt pair is somewhat sensitive to environmental conditions, which caused a consistent shift in the curves of each series with respect to each other.

Unlike Pd─Pt, the curve for Pt─Co exhibits a more symmetric shape with a smaller scatter for most *F*_Pt_ values (Figure 2b). For Pd─Co, at low Pd fractions, the curve shows a sharper increase in *F*_Pd_ compared to Pt─Co, and a second step increase at *f*_Pd _= 0.5 (Figure 2c). This system also exhibits larger amounts of absorbed Pd than the feed (i.e., *F*_Pd_ > *f*_Pd_) for most feed compositions. The majority of absorbed Pd in the palladium‐containing pairs (Pd─Pt and Pd─Co) suggests that the Pd precursors have a higher tendency than Pt and Co precursors to impregnate the P2VP domains. Yet, the different shapes of the curves point to fundamental differences in the behaviors of these metal pairs, and suggest that additional factors are at play rather than merely different binding affinities. Using reduced variables such as the molar fraction values is apparently insufficient to explain these differences.

### Insights from the Analysis of Individual Metal Concentrations

**Figure 3:**
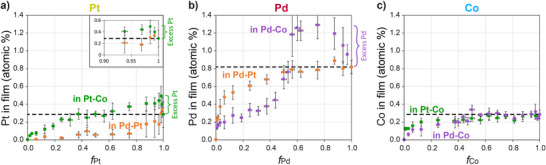
Atomic concentrations of a) Pt, b) Pd, and c) Co, as measured by EDX, as a function of their molar fraction in the feed solution. Each set of data points corresponds to the amount of one of the metals in a certain bimetallic pair. Error bars denote the standard deviation from 3 to 11 sample repetitions. The value at *f* = 1 is marked with a dashed line for visual reference; metal absorbed in excess above this value is denoted (applies to Pt and Pd). The inset in a) highlights the behavior at the high *f*_Pt_ limit.

To better understand the factors behind the different behaviors of each metal pair, we looked at the amount of each metal in the pair (i.e., its atomic concentrations in the block copolymer film used for calculating the metal mole fractions discussed above) as measured by EDX (Figure 3). Although the values depend on the electron beam‘s accelerating voltage and instrument geometry, the trends observed for a certain metal precursor with respect to its feed composition can be used to compare its behavior with the two other metal precursors with which it is used to impregnate the film. The amounts of Pt and Pd in the Pd─Pt system (orange datasets in Figure 3a,b, respectively) show a monotonic increase of the absorbed precursor with its increasing concentration in the feed solution, reaching the maximum (“saturation”) value at *f* = 1 (where the impregnation solution is purely monometallic). This behavior is expected and reflects a competition of both metals on the binding sites, where the maximum absorbed amount of the metal of interest is obtained when the impregnation solution contains only that metal precursor. The respective shapes of the curves formed by the orange datasets (a fast increase that reaches saturation for Pd in Figure 3b, and a gradual increase of the Pt curve with a sharp rise at *f*_Pt_ → 1 in Figure 3a) and the higher maximum concentration of Pd compared to that of Pt (more than x2.5) both indicate easier binding of the Pd precursors to pyridine.^[^ ^44^ ^]^ This behavior is reflected in the curve of *F*_Pd_ in the Pd─Pt case (Figure 2a), which features *F*_Pd_ > 0.5 for most feed compositions (even at extremely low *f*_Pd_), and a resulting composition that exceeds the feed (*F*_Pd_ > *f*_Pd_) across its entire composition range. Lastly, the rather low atomic concentrations of Pt and their large relative error (Figure 3a, orange points) provide another explanation for the large scatter in *F*_Pd_ observed in the curve of Pd─Pt (Figure 2a).

The curves representing the Co precursor in both its pairs (Pt─Co and Pd─Co, Figure 3c) largely overlap and show a monotonic increase with increasing *f*_Co_. However, the amounts for Pt and Pd in their pairs with Co (Figure 3a,b, green and purple datasets, respectively) follow a completely different trend compared to those discussed thus far. Above *f*_Pt_ ≈ 0.6 and *f*_Pd_ ≈ 0.5 feed composition, the resulting impregnated concentrations of both metal precursors increase beyond the level obtained for them in the corresponding purely monometallic feed experiments (i.e., at *f* = 1), reaching a maximum level at *f*_Pt_ = 0.94 and at *f*_Pd_ = 0.75 before dropping back down toward the composition of the purely monometallic feed (*f* = 1). This behavior indicates that at certain feed composition ranges in the Pt─Co and Pd─Co pairs, the impregnated polymer contains more Pt or Pd (respectively) than it holds when impregnated with this metal alone. This result points at a cooperative mechanism between the Co complex and the other metal precursor in both Pt─Co and Pd─Co pairs, that occurs in parallel to the competition over binding sites (as will be shown below).

### Molecular‐Level Insights on the Polymer‐Metal Interaction from a Model System

The behaviors elucidated from the EDX quantification of the amounts of each metal in the co‐impregnation solutions invoked the need to fundamentally understand the nature of the polymer‐metal interactions in the different bimetallic systems. Insights into this aspect were obtained by employing the water‐soluble 2‐methylpyridine (2‐picoline) to model the repeat unit of P2VP. The total concentration of the different metal precursors in D_2_O was equal to the concentration of the impregnation solutions (i.e., 2 mM). To simulate the abundance of pyridine groups available in the PS‐*b*‐P2VP film during impregnation, the concentration of 2‐picoline was set to 16 mM (8 metal precursor equivalents). Additionally, an equimolar concentration of HCl (16 mM) was used to fully protonate the 2‐picoline (termed “2‐picolinium” hereafter) to adhere to the impregnation conditions without creating an excess of free protons.

For the 2‐picolinium solutions containing pairs of metal precursors, we originally tested mixtures with equal concentrations of each metal (i.e., 1 mM or *f* = 0.5). However, the Pd─Pt solution with 2‐picolinium was unstable and formed a yellow precipitate over time, so we had to reduce the *f*_Pd_ to 0.13 to achieve a stable solution. The Pd─Co solution with 2‐picolinium precipitated immediately at all compositions tested, which is likely related to the cooperative mechanism inferred previously.

**Figure 4:**
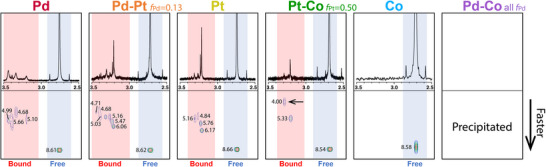
DOSY‐NMR spectra of solutions containing 16 mM of 2‐picoline, 16 mM of HCl, and 2 mM of different metal precursors. The spectra show the 2.5–3.5 ppm portion of the spectrum corresponding to the methyl group peaks of the free 2‐picolinium (blue) and metal‐bound 2‐picoline (red) species. The diffusion coefficient values are denoted next to each peak in units of 10^−10^ m^2^s^−1^. The arrow points to the peak attributed to a picoline‐Pt─Co species.

The ^1^H‐NMR spectra of all solutions (Figure S4) exhibited a set of peaks attributed to the excess of unbound 2‐picolinium (termed as the “free species”). The spectra of all the solutions containing Pd or Pt precursors (including pairs consisting of any of these precursors) displayed additional sets of peaks with slightly different chemical shifts. These peaks appeared after only a few minutes of incubation in the PdCl_4_^2–^ solution, whereas all the solutions containing the PtCl_4_^2–^ required 24 h for additional peaks to appear, and four days to fully reach equilibrium (Figure S5). Diffusion‐ordered NMR spectroscopy (DOSY‐NMR; Figure 4) of these solutions showed that the species associated with the additional sets of peaks have lower self‐diffusion coefficients (in the range of (4.0–6.2) × 10^−10^ m^2^s^−1^) than those of the free species (∼8.6 × 10^−10^ m^2^s^−1^) which indicates that their effective hydrodynamic radii are larger than that of the unbound 2‐picolinium. Hence, we assigned the additional sets of peaks to the respective 2‐picoline‐metal complexes (termed as the “bound species”).^[^ ^45^ ^]^

**Figure 5:**
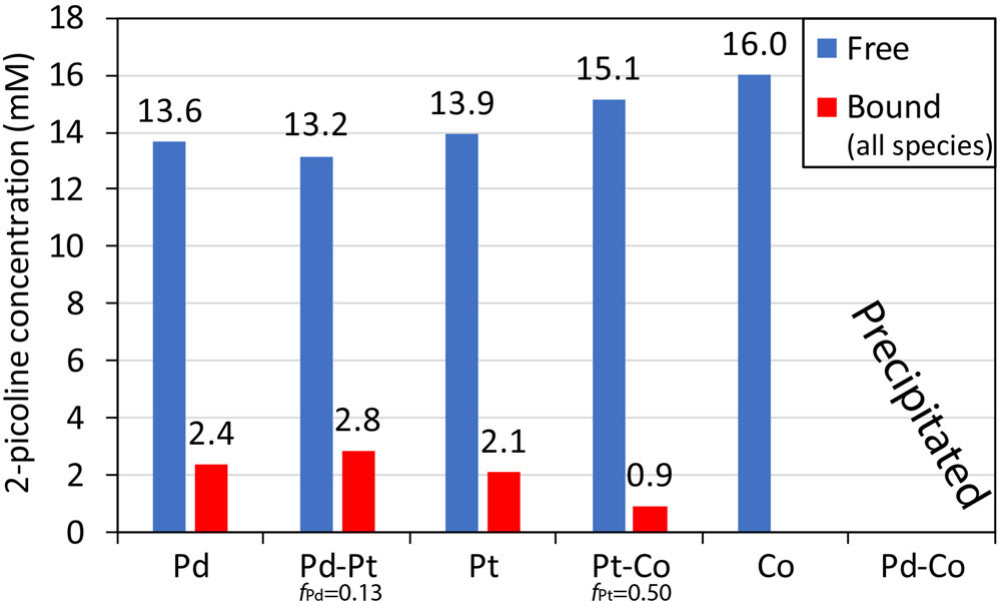
The concentrations of free 2‐picolinium (blue) and metal‐bound 2‐picoline (red) species in solutions containing 2 mM metal precursors, 16 mM 2‐picoline, and 16 mM HCl.

The concentrations of the free and bound species were quantified through the peak integration of the respective methyl groups (Figure 5). The solutions containing PdCl_4_^2–^, PtCl_4_^2–^, and a mixture of the Pd─Pt precursors featured similar concentrations of the free species as well as similar concentrations of the bound species. The DOSY‐NMR results displayed similar behavior between the bound species as well, where their self‐diffusion coefficients spanned the range of (4.7–6.2) × 10^−10^ m^2^s^−1^ (Figure 4). This suggests that the Pd─Pt solution contained a mixture of similar types of Pd‐picoline and Pt‐picoline complexes.

Interestingly, solutions containing only Co(CN)_6_^3–^ with 2‐picoline and HCl exhibited only the set of peaks associated with the free species. This implies that the Co complex does not bind to the pyridine ring of the 2‐picoline through a coordinative bond. The absorption of Co precursor in the polymer film during the impregnation process is thus explained by the electrostatic interactions between the negatively charged Co(CN)_6_^3–^ complex and the positive pyridinium groups in the P2VP. The self‐diffusion coefficient of 2‐picolinium in its solution with the Co precursor is similar to that of the other free species (Figure 4), suggesting that the 2‐picolinium and the Co(CN)_6_^3–^ complex exist as free ions in solution. In the solution containing even amounts of the Pt and Co precursors (*f*_Pt _= 0.50), only Pt could partake in metal‐ligand coordination with the 2‐picoline, and thus the concentration of the bound species was only 0.90 mM, which is nearly half that of the concentrations of the bound species in the other solutions.

The DOSY‐NMR data of the Pt─Co system (Figure 4) led to an unexpected observation. Apart from the peak attributed to the free species (with *D* = 8.54 × 10^−10^ m^2^s^−1^), two main peaks representing the bound species are observed: one that is attributed to the Pt‐picoline complex (with *D* = 5.33 × 10^−10^ m^2^s^−1^) and another one featuring a distinctly low self‐diffusion coefficient (*D* = 4.00 × 10^−10^ m^2^s^−1^), which indicates a larger hydrodynamic radius than that of the Pt‐picoline complex. This observation is explained by the known tendency of cyanide ligands to displace chloride ligands in the PdCl_4_^2–^ and PtCl_4_^2–^ complexes and coordinate to the metal center through their nitrogen atom (see Figure S6).^[^ ^46^ ^]^ Hence, we attribute the slower‐diffusion peak to a [2‐picoline‐PtCl_2_‐NC‐Co(CN)_5_]^3–^ bimetallic complex, where a cyanide ligand bridges between the Pt and the Co centers. In the case of the solution of Pd and Co precursors with 2‐picolinium, the observed precipitation can be understood as resulting from the formation of an insoluble extended network.^[^ ^46^ ^]^

The observation that the introduction of the Co precursor enhances the absorption of the Pd and Pt precursors and not the other way around draws the following pictures regarding the impregnation process. Initially, all metal complexes are drawn into the protonated P2VP domains by electrostatic attraction. Over time, the Pd and Pt precursors form coordination bonds with the pyridine groups. The formation of pyridine‐Pd bonds is favorable over the formation of pyridine‐Pt bonds,^[^ ^44^ ^]^ hence *F*_Pd_ > *f*_Pd_ in the Pd─Pt impregnation experiments (Figure 2a). In the Co‐containing pairs, the presence of the Co(CN)_6_^3–^ complexes creates additional binding sites for the Pd and Pt precursors using their coordination to the cyanide ligands. This cooperative effect thus allows the polymer film to absorb more Pd and Pt than in the absence of Co complexes (Figure 3a, green dataset and Figure 3b, purple dataset).

Further support for the picture evolving from the above experiments was obtained by performing the co‐impregnation experiments in acid‐free feeds. Under these conditions, the pyridine groups of the P2VP domains are unprotonated, which eliminates the ability of the polymer to attract the negatively charged precursors by electrostatic interactions. Indeed, impregnation with Co(CN)_6_^3–^ showed no absorbed Co. Nonetheless, the neutral pyridine groups were available for coordinative binding with the Pd and Pt precursors without being blocked from this type of binding by the protonation. In the case of Pd─Pt, the resulting impregnation dataset is less asymmetric compared to that obtained in under acidic conditions (Figure S7a). This suggests that the pyridine protonation created a higher barrier for Pt binding than for Pd binding. In the case of Co‐containing pairs, the *F*_Pd_ and *F*_Pt_ datasets were higher than the curves of the acidic impregnation (Figure S7b,c). From the Co perspective, however, the situation is reversed: in the acidic impregnation the presence of the absorbed Co(CN)_6_^3–^ attracted an excess of Pd and Pt precursors, but in the acid‐free impregnation it is the presence of pyridine‐coordinated Pd and Pt precursors that attracted the absorption of the Co precursors (through the cyanide bridging). This is manifested by the non‐zero Co concentrations in the Pt─Co and Pd─Co impregnation experiments (Figure S8c), which is considered to be an excess of absorbed Co, considering that the Co(CN)_6_^3–^ alone did not impregnate the film at all (*F*_Co_ = 0 at *f*_Co_ = 1).

### An Empirical Model for the Co‐Impregnation

The results so far unravelled two mechanisms of co‐impregnation: competitive co‐impregnation (in all three systems) and cooperative co‐impregnation (in the Pd─Co and Pt─Co systems). We strived to model the behaviors of acidic co‐impregnations portrayed by the datasets in Figure 2 in order to provide a predictive aid for designing the composition of bimetallic NWs prepared by the block copolymer‐mediated approach.

The co‐impregnation process resembles free radical copolymerization in a few aspects. Both processes give rise to product composition that depends on the feed composition in a non‐trivial way. Additionally, the shapes of the curves shown in Figure 2a,b resemble the typical behaviors of certain free radical copolymerization cases (i.e., Pd─Pt to the common case in free‐radical copolymerization and Pt─Co to azeotropic copolymerization).^[^ ^47^ ^]^ Lastly, the concept of reactivity ratios used in the Mayo‐Lewis equation for free radical copolymerization,^[^ ^47^, ^48^ ^]^ which relates the composition of the resulting copolymer to the reactivity of the active chain ends and the monomers, seems relevant to the co‐impregnation process. Although we could not strike a direct analogy between the kinetics of free radical copolymerization and the co‐impregnation process, we were inspired by the idea that the co‐impregnation behavior may also depend on parameters that relate to the affinity of each metal precursor to the P2VP and are also influenced by the other metal. For example, the cooperative impregnation mechanism found for the Pt─Co and Pd─Co pairs enables two modes of binding for Pd and Pt precursors (i.e., to the pyridine group and the cyanide ligands of absorbed Co), which resembles the addition of comonomer A to the two types of reactive chain ends of the propagating chains in free radical copolymerization.

Fitting the datasets shown in Figure 2 to the Mayo–Lewis equation gave mediocre results (see discussion and Figure S9). However, empirical modifications applied to the Mayo‐Lewis expression (see Supporting Information for discussion) resulted in Equation (1), which provided excellent fits to the three datasets of Pd─Pt, Pt─Co, and Pd─Co pairs (curves in Figure 2):

(1) $$\begin{array}{cc} F_{A} & =\alpha \frac{r_{A}^{+}f_{A}+\sqrt{f_{A}f_{B}}}{r_{A}^{+}f_{A}+2\sqrt{f_{A}f_{B}}+r_{B}^{+}f_{B}}\\ & \;+\left(1-\alpha \right)\frac{r_{A}^{-}f_{A}-\sqrt{f_{A}f_{B}}}{r_{A}^{-}f_{A}-2\sqrt{f_{A}f_{B}}+r_{B}^{-}f_{B}} \end{array}$$

This equation comprises two modified Mayo–Lewis‐like expressions, which differ by the sign of the $\sqrt{f_{A}f_{B}}$ term (and are denoted as the “positive” and “negative” expressions hereafter). Each expression includes parameters *r*_A_ and *r*_B_ that relate to each metal, with fraction *α* of the positive expression. Table 1 shows the fitting parameters that were determined for each metal pair by fitting each dataset to Equation (1).

**Table 1:** Parameters obtained from fitting the data in Figure 2 to Equation (1).

	$r_{\mathrm{Pd}}^{+}$	$r_{\mathrm{Pt}}^{+}$	$r_{\mathrm{Co}}^{+}$	$r_{\mathrm{Pd}}^{-}$	$r_{\mathrm{Pt}}^{-}$	$r_{\mathrm{Co}}^{-}$	α
Pd─Pt	7.23	0.06	–	–	–	–	1
Pt─Co	–	0.34	0.50		2.76	0.55	0.91
Pd─Co	1.89	–	0.11	1.04	–	1.04	0.93

The fitting showed that for the Pd─Pt case, only the positive expression applies (*α* = 1). Hence, we attribute the “positive” expression to the competitive co‐impregnation mechanism, and the *r* ^+^ parameters to the respective binding affinities of each metal precursor to the polymer film in the presence of the other metal. Indeed, the *r* ^+^ values determined for each metal are not intrinsic values, and depend on its counterpart (in analogy to the reactivity ratios in free radical copolymerization). The Pd precursor displays the highest affinity values to the polymer, which is reflected in the larger values of *r*_Pd_^+^ compared to *r*_Pt_^+^ and *r*_Co_^+^ in their respective pairs.

**Figure 6:**
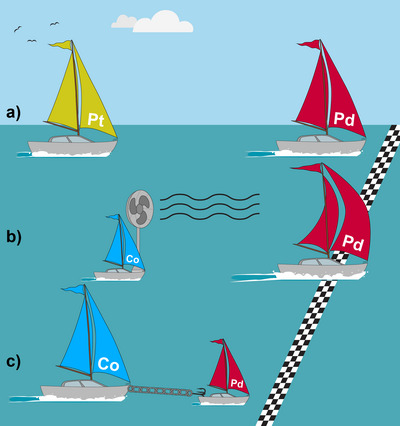
Analogy of the three co‐impregnation mechanisms to a boat race, where the feed fraction of each metal precursor is represented by the size of its boat, and the relative locations of the boats in the race represent the resulting composition. a) Competitive co‐impregnation, where one metal precursor is absorbed more into the polymer (illustrated as the faster boat). b) Cooperative co‐impregnation, in which the Co precursor at low Co feed fractions (high *f*_Pd_) facilitates the absorption of the other metal (illustrated by the fan on the Co boat that blows wind into the Pd sails). c) The hindering effect, in which Co precursors, at high Co feed fractions (low *f*_Pd_), inhibit the absorption of the other metal into the polymer film (illustrated by a chain that slows down the Pd boat).

The values determined for the fraction *α* for the Pt─Co and Pd─Co cases show that the contribution of the “negative” expression to the curve is much smaller than that of the “positive” expression. The “negative” expression may be associated with the cooperative co‐impregnation effect observed in the Pt─Co and Pd─Co systems at high *f*_Pd_ and *f*_Pt_ limit. Deeper analysis of the “negative” expression (see discussion and Figure S11) reveals that the cooperative effect in Pt─Co takes place at a larger feed range than in the Pd─Co pair, albeit with lower magnitude. The larger cooperative effect for Pd─Co fits the overall trend of the favorable coordination of Pd, not only to the pyridine groups, but also to the cyanide ligands of the Co precursor. However, at the low *f*_Pd_ limit, the “negative” expression reflects steric and electrostatic hindrance effects, which actually *lower* the relative amount of absorbed Pd precursor compared to the Pd─Pt pair (see Figure 3b). The different contributors to the impregnation process, i.e., the competitive, cooperative, and hindering effects, are illustrated in Figure 6.

## Conclusions

This work unraveled the fundamental principles that govern the impregnation of block copolymer films with metal precursors, which enables full predictability and control over the desired composition of bimetallic nanowires formed after plasma treatment. Although electrostatic attraction is the initial driving force for the absorption of the metal precursors into the film, the ability of some of these precursors to form coordination bonds with the polymer functionalities gives rise to two modes of mutual influence. The first mode, which applies to all metal pairs (and is thus believed to be general to any pair of metal precursors), is competition over the binding sites in the polymer and reflects an indirect mutual influence. The second mode, which is relevant only to certain metal pairs based on their chemistry, is direct interaction between the metal precursors. In this mode, the absorption of one metal precursor may either facilitate the absorption of the other metal precursor (even beyond the saturation amount for this metal precursor alone), or may hinder it, depending on the feed composition. With other pairs of metal complexes, which feature additional types of metal‐ligand chemistries, it is possible that additional impregnation behaviors will be observed.

This study adds the composition dimension to the already highly versatile approach for nanowire fabrication using block copolymer films. This addition will greatly extend its applicability; virtually an endless number of compositions and morphologies of bimetallic structures are now accessible through the rational design of components and economic, highly scalable assembly/fabrication processes. These include periodic arrays of bimetallic dots, macroscopically‐oriented bimetallic nanowires and meshes, and their combinations. The accessibility of highly organized arrays of bimetallic nanoscale components opens up new horizons for the fabrication of advanced photonic devices such as meta‐surfaces, sensors, integrated circuits, as well as sophisticated magnetic and electrocatalytic devices, and solar and energy applications.

## Conflict of Interests

The authors declare no conflict of interest.

## Supporting information



Supporting Information

## Data Availability

The data that support the findings of this study are available in the Supporting Information of this article.

## References

[1] J. Jiu , K. Suganuma , IEEE Trans. Compon. Packag. Manuf. Technol. 2016, 6, 1733–1751.

[2] H. Wei , D. Pan , S. Zhang , Z. Li , Q. Li , N. Liu , W. Wang , H. Xu , Chem. Rev. 2018, 118, 2882–2926. 29446301 10.1021/acs.chemrev.7b00441

[3] M. Schvartzman , D. Tsivion , D. Mahalu , O. Raslin , E. Joselevich , Proc. Natl. Acad. Sci. USA. 2013, 110, 15195–15200. 23904485 10.1073/pnas.1306426110 PMC3780857

[4] R. A. Pala , J. White , E. Barnard , J. Liu , M. L. Brongersma , Adv. Mater. 2009, 21, 3504–3509.

[5] T. Sannicolo , M. Lagrange , A. Cabos , C. Celle , J. P. Simonato , D. Bellet , Small 2016, 12, 6052–6075. 27753213 10.1002/smll.201602581

[6] C. Rousse , P. Fricoteaux , J. Mater. Sci. 2011, 46, 6046–6053.

[7] K. V. Frolov , M. A. Chuev , I. S. Lyubutin , D. L. Zagorskii , S. A. Bedin , I. V. Perunov , A. A. Lomov , V. V. Artemov , D. N. Khmelenin , S. N. Sulyanov , J. Magn. Magn. Mater. 2019, 489, 165415.

[8] L. Liu , E. Pippel , R. Scholz , U. Gosele , Nano Lett. 2009, 9, 4352–4358. 19842671 10.1021/nl902619q

[9] F. Kadirgan , A. M. Kannan , T. Atilan , S. Beyhan , S. Ozenler , S. Suzer , A. Yörür , Int. J. Hydrogen Energy 2009, 34, 9450–9460.

[10] R. Ferrando , J. Jellinek , R. L. Johnston , Chem. Rev. 2008, 108, 845–910. 18335972 10.1021/cr040090g

[11] B. Lim , M. Jiang , P. H. Camargo , E. C. Cho , J. Tao , X. Lu , Y. Zhu , Y. Xia , Science 2009, 324, 1302–1305. 19443738 10.1126/science.1170377

[12] Y. Liu , M. Chi , V. Mazumder , K. L. More , S. Soled , J. D. Henao , S. Sun , Chem. Mater. 2011, 23, 4199–4203.

[13] H. Lv , Y. Wang , A. Lopes , D. Xu , B. Liu , Appl. Catal., B 2019, 249, 116–125.

[14] F. Chang , Z. Bai , M. Li , M. Ren , T. Liu , L. Yang , C.‐J. Zhong , J. Lu , Nano Lett. 2020, 20, 2416–2422. 32046493 10.1021/acs.nanolett.9b05123

[15] S. T. Christensen , H. Feng , J. L. Libera , N. Guo , J. T. Miller , P. C. Stair , J. W. Elam , Nano Lett. 2010, 10, 3047–3051. 20698618 10.1021/nl101567m

[16] M. B. Cortie , A. M. McDonagh , Chem. Rev. 2011, 111, 3713–3735. 21235212 10.1021/cr1002529

[17] G. J. Hutchings , C. J. Kiely , Acc. Chem. Res. 2013, 46, 1759–1772. 23586905 10.1021/ar300356m

[18] S. Duan , R. Wang , Prog. Nat. Sci.: Mater. Int. 2013, 23, 113–126.

[19] M. R. Bockstaller , Y. Lapetnikov , S. Margel , E. L. Thomas , J. Am. Chem. Soc. 2003, 125, 5276–5277. 12720430 10.1021/ja034523t

[20] E. C. Walter , B. J. Murray , F. Favier , R. M. Penner , Adv. Mater. 2003, 15, 396–399.

[21] Z. Meng , G. Li , S. C. Yiu , N. Zhu , Z. Q. Yu , C. W. Leung , I. Manners , W. Y. Wong , Angew. Chem. Int. Ed. 2020, 59, 11521–11526. 10.1002/anie.202002685 32243037

[22] C. Zhang , S. N. Oliaee , S. Y. Hwang , X. Kong , Z. Peng , Nano Lett. 2016, 16, 164–169. 26642094 10.1021/acs.nanolett.5b04518

[23] F. Cai , L. Yang , S. Shan , D. Mott , B. H. Chen , J. Luo , C.‐J. Zhong , Catalysts 2016, 6, 96.

[24] R. K. Ramachandran , J. Dendooven , M. Filez , V. V. Galvita , H. Poelman , E. Solano , M. M. Minjauw , K. Devloo‐Casier , E. Fonda , D. Hermida‐Merino , ACS Nano 2016, 10, 8770–8777. 27585708 10.1021/acsnano.6b04464

[25] W. B. Jung , S. Y. Cho , B. L. Suh , H. W. Yoo , H. J. Jeon , J. Kim , H. T. Jung , Adv. Mater. 2019, 31, 1805343. 10.1002/adma.201805343 30549106

[26] N. L. Netzer , C. Qiu , Y. Zhang , C. Lin , L. Zhang , H. Fong , C. Jiang , Chem. Commun. 2011, 47, 9606. 10.1039/c1cc13641k 21796309

[27] R. MacKenzie , C. Fraschina , T. Sannomiya , V. Auzelyte , J. Vörös , Sensors 2010, 10, 9808–9830. 22163441 10.3390/s101109808 PMC3231022

[28] M. J. Fasolka , A. M. Mayes , Annu. Rev. Mater. Res. 2001, 31, 323–355.

[29] A. Knoll , A. Horvat , K. S. Lyakhova , G. Krausch , G. J. A. Sevink , A. V. Zvelindovsky , R. Magerle , Phys. Rev. Lett. 2002, 89, 035501. 12144400 10.1103/PhysRevLett.89.035501

[30] J. Chai , D. Wang , X. N. Fan , J. M. Buriak , Nat. Nanotechnol. 2007, 2, 500–506. 18654348 10.1038/nnano.2007.227

[31] J. Chai , J. M. Buriak , ACS Nano 2008, 2, 489–501. 19206575 10.1021/nn700341s

[32] D. O. Shin , J. H. Mun , G.‐T. Hwang , J. M. Yoon , J. Y. Kim , J. M. Yun , Y.‐B. Yang , Y. Oh , J. Y. Lee , J. Shin , ACS Nano 2013, 7, 8899–8907. 24007296 10.1021/nn403379k

[33] K. G. A. Tavakkoli , S. M. Nicaise , K. R. Gadelrab , A. Alexander‐Katz , C. A. Ross , K. K. Berggren , Nat. Commun. 2016, 7, 10518. 26796218 10.1038/ncomms10518 PMC4736107

[34] M. Ma , C. A. Ross , ACS Appl. Polym. Mater, 2024, 6, 11626–11632.

[35] J. Y. Kim , B. H. Kim , J. O. Hwang , S. J. Jeong , D. O. Shin , J. H. Mun , Y. J. Choi , H. M. Jin , S. O. Kim , Adv. Mater. 2013, 25, 1331–1335. 23239284 10.1002/adma.201204131

[36] R. Liu , H. Huang , Z. Sun , A. Alexander‐Katz , C. A. Ross , ACS Nano 2021, 15, 16266–16276. 34647737 10.1021/acsnano.1c05315

[37] J. H. Mun , S. K. Cha , Y. C. Kim , T. Yun , Y. J. Choi , H. M. Jin , J. E. Lee , H. U. Jeon , S. Y. Kim , S. O. Kim , Small 2017, 13, 1603939. 10.1002/smll.201603939 28218488

[38] Z. Liu , T. Chang , H. Huang , T. He , ACS Appl. Mater. Interfaces 2015, 7, 25938–25945. 26517409 10.1021/acsami.5b08751

[39] O. Burg , R. A. Sanguramath , E. Michman , N. Eren , I. Popov , R. Shenhar , Soft Matter 2021, 17, 9937–9943. 34693421 10.1039/d1sm01313k

[40] J. H. Mun , Y. H. Chang , D. O. Shin , J. M. Yoon , D. S. Choi , K. M. Lee , J. Y. Kim , S. K. Cha , J. Y. Lee , J. R. Jeong , Y. H. Kim , S. O. Kim , Nano Lett. 2013, 13, 5720–5726. 24083558 10.1021/nl403542h

[41] A. K. Taylor , D. S. Perez , X. Zhang , B. K. Pilapil , M. H. Engelhard , B. D. Gates , D. A. Rider , J. Mater. Chem. A 2017, 5, 21514–21527.

[42] T. B. Massalski , H. Okamoto , P. Subramanian , L. Kacprzak , W. W. Scott , Binary alloy phase diagrams, Vol. 2, American Society for Metals, Metals Park, OH, 1986.

[43] The NWs made of Pd‐Co are less uniform compared to Pd‐Pt and Pt‐Co, due to a difference in the internal structure of these NWs, despite having the same fabrication process and building blocks. Additionally, all STEM images and EDX maps show specks of metal scattered between the NWs, which are a result of a P2VP wetting layer in the polymer film due to the preferable wetting of the SiOx substrate with the P2VP component.

[44] F. Basolo , H. B. Gray , R. G. Pearson , J. Am. Chem. Soc. 1960, 82, 4200–4203.

[45] The NMR spectra show a few peaks for the methyl groups of the bound species even in the monometallic precursor solutions. This suggests that several types of complexes exist, which may contain different numbers of 2‐picoline ligands (up to four). A thorough analysis is required to distinguish between these types of complexes, but this is beyond the scope of this paper.

[46] B. W. Pfennig , A. B. Bocarsly , R. K. Prud'homme , J. Am. Chem. Soc. 1993, 115, 2661–2665.

[47] R. J. Young , P. A. Lovell , Introduction to Polymers, Vol. 3 ^rd^, CRC Press, Boca Raton, FL, 2011.

[48] F. R. Mayo , F. M. Lewis , J. Am. Chem. Soc. 1944, 66, 1594–1601.

