# Supplementary material for: Toward Bimetallic Nanowire Arrays with Controlled Compositions Using Block Copolymer Films: The Interplay Between Metal Precursors
Source: Angew Chem Int Ed Engl. 2025 Aug 29;64(43):e202512695. doi: 10.1002/anie.202512695 (PMC12535391; doi:10.1002/anie.202512695)
Supplement: Supplementary file 1 — Supporting Information [file ANIE-64-e202512695-s001.pdf]

Toward Bimetallic Nanowire Arrays with Controlled Compositions Using Block Copolymer Films: The Interplay Between Metal Precursors

Ofer Burg, Carmel Cohen, and Roy Shenhar*

[*] O. Burg, C. Cohen. Prof. Dr. R. Shenhar
The Institute of Chemistry and the Harvey M. Krueger Family Center for Nanoscience and Nanotechnology
The Hebrew University of Jerusalem
Jerusalem 9190401, Israel
E-mail: roys@huji.ac.il

Supporting Information

Experimental Section

Materials

Na_2PdCl_4 (TCL), K_2PtCl_4 (Sigma-Aldrich), $K_3Co(CN)_6$ (Strem Chemicals), D_2O (Zeochem), 2-picoline (Alfa Aesar), PS-*b*-P2VP (M_n 188 kg mol^{-1}, 71.8 %wt PS, polydispersity index 1.18, Polymer Source, Inc.) were used as received.
Ultrapure water with resistivity 18.2 MΩ·cm from Barnstead™ GenPure™ Pro water purification system was used for this work

Sample preparation

Silicon wafers with 100 nm-thick oxide layer were cleaned using piranha solution. Block copolymer films were prepared by spin coating 0.28 wt% chloroform solutions of the polymer at 3,000 RPM for 30 s. The films were then annealed in saturated chloroform vapor for 20 min. Each sample was cut into 16-24 pieces for separate use.
For the different co-impregnation (feed) solutions, 20 mM metal precursor solutions of Na_2PdCl_4, K_2PtCl_4, and $K_3Co(CN)_6$ were prepared in ultrapure water and mixed at different ratios. The solutions were then mixed with 1 wt% HCl to achieve a total metal concentration of 2 mM and 0.9 wt% HCl. The polymer-coated substrates were immersed for 24 h in the feed solution, washed with deionized water, and dried under a nitrogen stream.
After impregnation, each film was separated from the substrate by inserting the substrate into a 10 vol% HF solution. The floated film was then transferred onto a TEM substrate. Copper grids were used for polymer film characterization; carbon-coated copper grids and silicon nitride membrane windows were used for NW characterization.
Oxygen plasma was performed using Jupiter III parallel plate reactive ion etcher (March Plasma Systems), operating at 60 mTorr of O_2 at 40 W power for 45 s, followed by Ar plasma at 0.15 mbar at 100 W power for 6 s using PICO low-pressure plasma system (Diener electronic).

Sample Characterization

Scanning transmission electron microscopy (STEM) images of polymer films were acquired using an Analytical HR-SEM Apreo 2S (Thermo Fisher Scientific) working in STEM mode at an accelerating voltage of 20 kV. Energy Dispersive X-ray Spectroscopy (EDX) quantification of films was performed with an UltraDry Premium 60 mm^2 Silicon Drift detector. High resolution TEM and STEM imaging of metallic nanowires were done with (S)TEM Talos F200i (Thermo Fisher Scientific) operated at 200 kV. The EDX mapping was performed in STEM mode with a Dual-XFlash 6 EDS system.
^{1}H-NMR measurements were performed on a 400 MHz NEO AVANCE Bruker spectrometer. Diffusion-ordered NMR was measured using a 500 MHz NEO Avance Bruker spectrometer equipped with a *Z*-gradient coil with a maximum field strength of 17 Tm^{-1}.
UV-vis spectra were collected using an Ocean Optics Red Tide USB650 Fiber Optic Spectrometer.

Data Analysis

Composition data was collected by quantification of the EDX spectra using Pt M, Pd L, and Co K lines. Each sample was measured at 3-6 different locations, and the atomic concentration of each metal and the metal fractions were averaged. Reported metal fractions and individual metal concentrations represent averages of the values determined independently from 3-11 samples that were prepared in the same way.

Extraction of the concentrations of the different 2-picoline species from the NMR data was done by normalizing the integration values of the peaks belonging to the methyl groups (2.7-2.8 and 3.1-3.4 ppm for free and bound species, respectively) to the total concentration of 2-picoline.
Fitting to equations and parameter extraction were performed using the curve fitting toolbox implemented in the Matlab software.

Substrate effect on the EDX spectrum

Samples prepared on silicon substrates exhibit a pronounced background from electron deceleration in the substrate (bremsstrahlung) and from excitation of Si emission lines (**Figure S1**a, blue spectrum). The peaks used for quantifying the composition of the Pd-Pt pair in the polymer film (the Pt M-line and Pd L-line at 2.05 keV and 2.84 keV, respectively) are close to the Si peak, and the added background greatly reduces the accuracy of the results.
Eliminating the silicon substrate by transferring the film to a copper TEM grid considerably reduced the background and increased the signal-to-noise ratio for the Pd and Pt peaks (**Figure S1**a, orange spectrum). Comparison of the film before and after the transfer (**Figure S1**b,c) confirms that it was unaffected by the process.

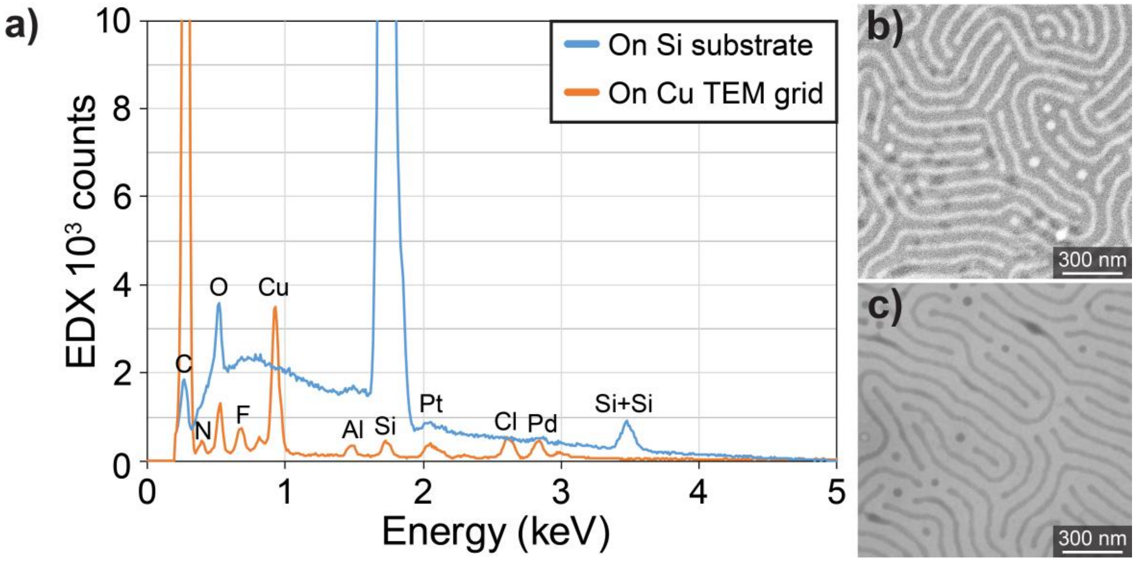

Figure S1. a) EDX spectra of a polymer film after co-impregnation with Pd-Pt, on a Si substrate (blue), and after being transferred onto a Cu grid (orange). Labels correspond to element assignment. b) SEM image of the film on a Si substrate. c) Bright field-STEM image of the film transferred onto a Cu TEM grid.

Composition retention after plasma

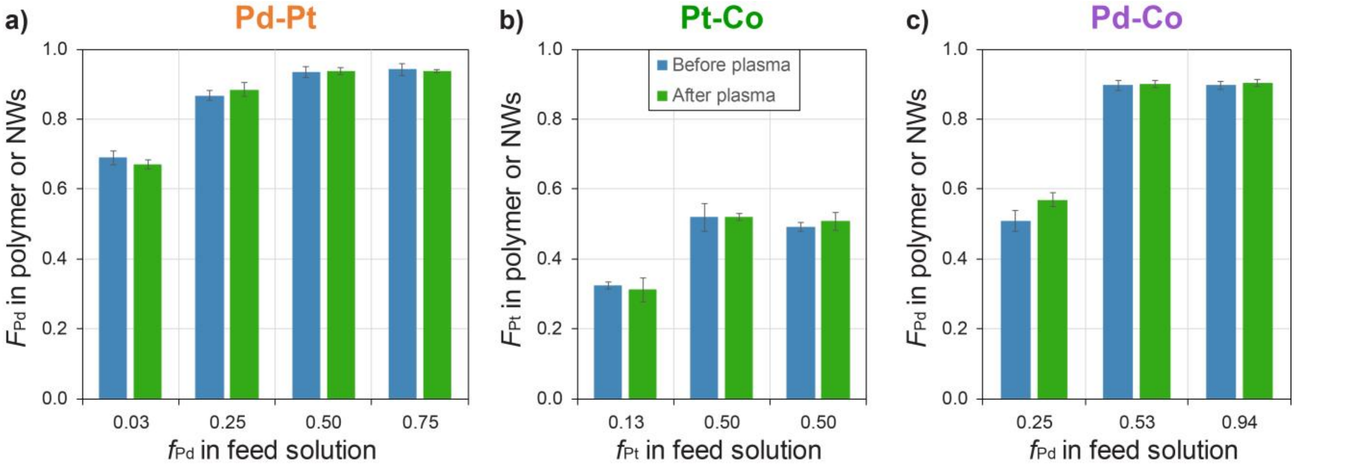

Figure S2. The metal fractions before plasma (blue) and after plasma (green) for samples of a) Pd-Pt, b) Pt-Co, c) Pd-Co.

Coherence of measurements within each series of experiments

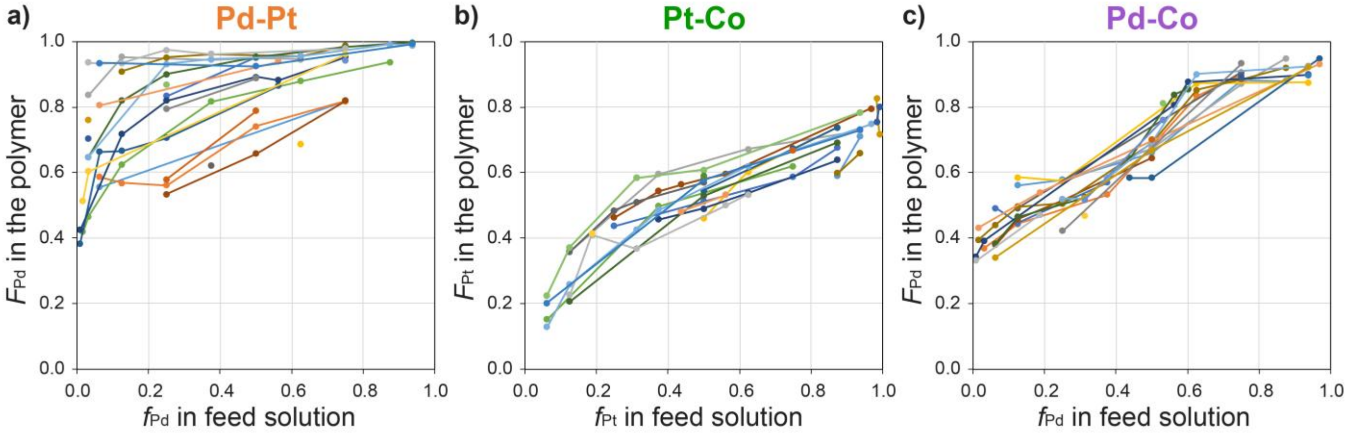

Figure S3. Curves of the individual experiments from which the data in **Figure 2** were averaged. Each colored series of data points represents a different set of samples that were prepared and measured together. Each data point represents an average taken from 3-6 locations on the sample. The error values of each measurement were negligible and were omitted for clarity. Lines were added as a guide to the eye.

NMR spectra of 2-picoline with the different metal precursors and their pairs

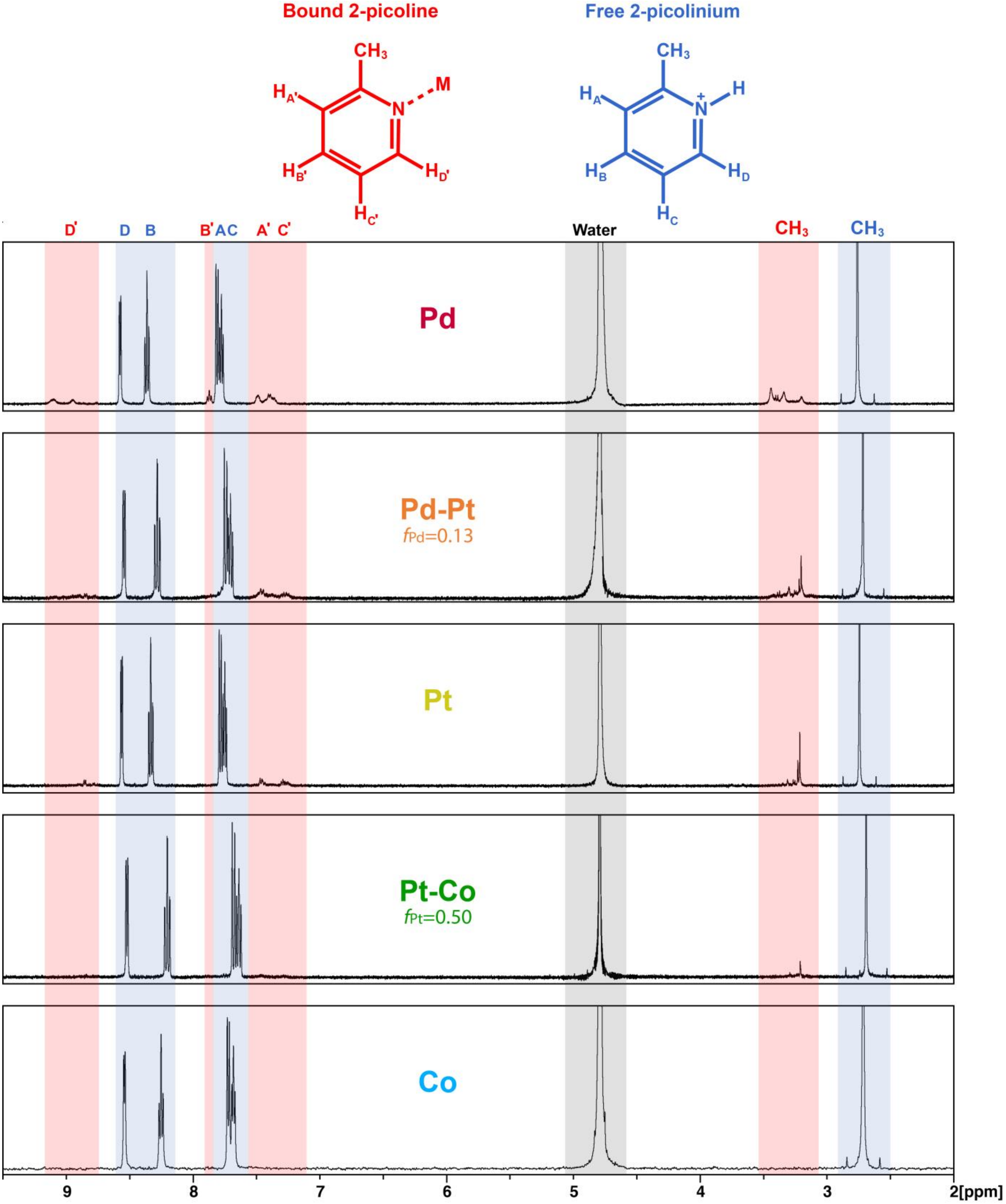

Figure S4. ^{1}H-NMR spectra of solutions containing 16 mM of 2-picoline, 16 mM of HCl, and 2 mM of different metal precursors. Peaks are marked for the hydrogens of the free 2-picolinium (blue) and metal-bound 2-picoline (red) species. Solutions containing the Pt precursor were measured after reaching equilibrium (four days).

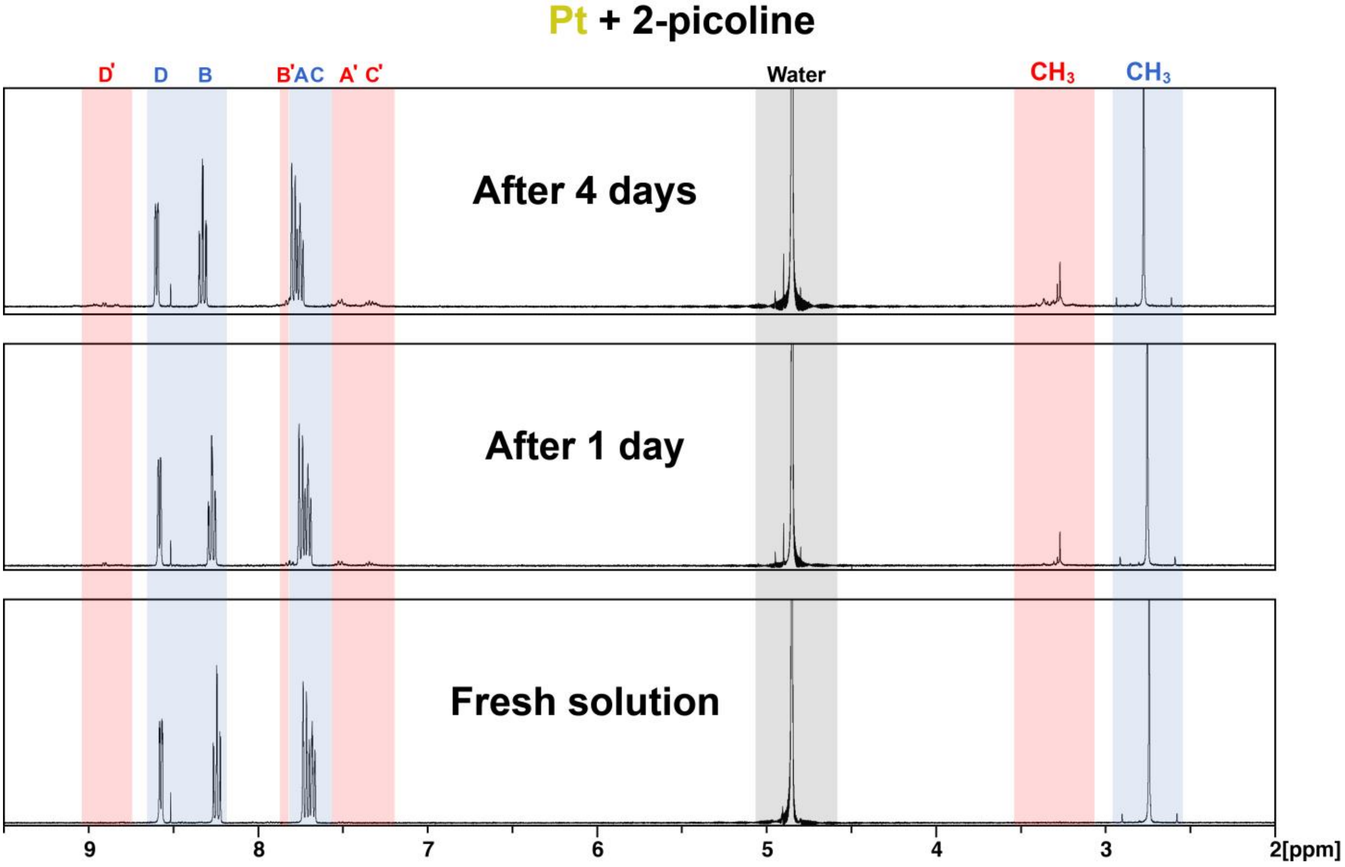

Figure S5. ^{1}H-NMR spectra of solutions containing 16 mM of 2-picoline, 16 mM of HCl, and 2 mM of K_2PtCl_4, measured immediately after preparation, after 1 day, and after 4 days. Peaks are marked for the free 2-picolinium (blue) and metal-bound 2-picoline (red) species.

UV-vis spectra of the Pd and Co complexes and their 3:1 mixture

Figure S6 shows the UV-vis spectra of acidic solutions of the Pd and Co complexes (red and blue curves, respectively), their sum (dashed purple curve, provided for comparison purposes), and the absorption spectrum of a 3:1 Pd:Co mixture after 24 h incubation (black curve). The solution of the mixture is identical to the solution used in the impregnation experiment that corresponded to the maximum cooperativity effect (feed composition f_{Pd}=0.75; see purple data in **Figure 3**b).

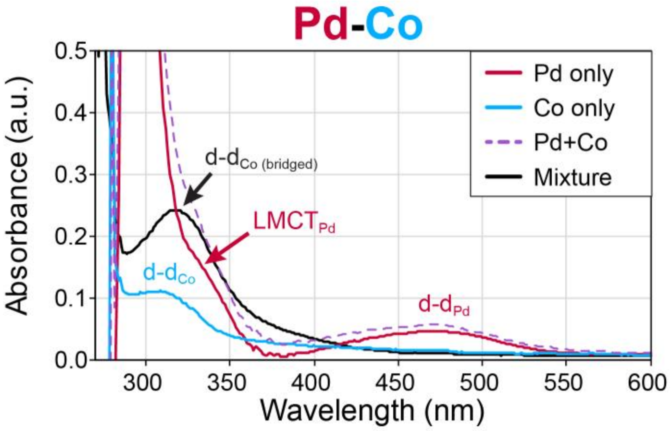

Figure S6. UV-vis absorption spectra of aqueous acidic solutions (HCl 0.9%) of the metal precursors Na_2PdCl_4 (red), $K_3Co(CN)_6$ (blue), and their mixture at a molar ratio of 3:1 Pd:Co after 24 h incubation (black). The sum of the spectra of the metal complexes is shown for comparison (purple dashed line). Labels denote the corresponding electronic transitions.

The absorption peak of $Co(CN)_6^{3-}$ at 310 nm (blue curve) corresponds to the d-d electronic transition $^1A_{1g}\rightarrow{}^1T_{1g}$.[1-2] Two peaks are observed for the $PdCl_4^{2-}$ complex (red curve), which are attributed to ligand-to-metal charge transfer (LMCT; shoulder at 340 nm) and the $^1A_{1g}\rightarrow{}^1A_{2g}$ d-d transition (at 470 nm).[3-4]

The spectrum of the mixture (black curve) exhibits a new absorption peak at 320 nm, and is thus distinctly different from the arithmetic sum of the two spectra of the separate metal complexes (dashed purple curve). This substantiates the conclusion that a reaction between the metal precursors took place, in which a Cl^- ligand in the Pd complex was displaced by the cyanide ligand of the $Co(CN)_6^{3-}$ complex, interacting through its nitrogen atom.[5] The 10 nm redshift of the Co d-d transition is consistent with binding of the cyanide ligands to Pd, which serves as a second back-bonding π-donor to the cyanide, causing the cyanide to become a weaker π-acceptor, which decreases the crystal field splitting. In addition, the ligand exchange in the Pd complex, from the weak field Cl^- to a strong field NC^-, increases the crystal field splitting of Pd and causes a blueshift of its absorption peaks. It is plausible to assume that the LMCT band of Pd shifts deeper into the UV range and the broad d-d transition overlaps the 320 nm peak of the bridged Co. Lastly, the increased intensity of the d-d transition of the bridged Co compared to the corresponding intensity of that transition in the separate Co complex is consistent with the reduced centrosymmetry of the Co complexes, which increases the intensity of the Laporte-forbidden d-d transition.

Acid-free co-impregnation experiments

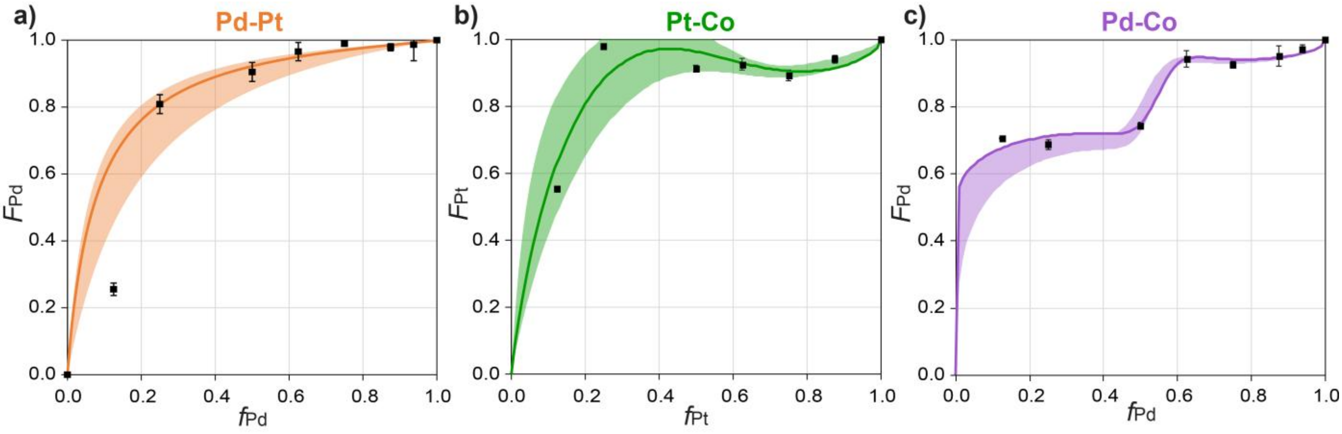

Figure S7. Dependence of the bimetallic composition inside the block copolymer film on the composition of the acid-free feed solution for each metal pair: a) Pd-Pt; b) Pt-Co; c) Pd-Co. The endpoints in each graph correspond to impregnation with a single metal. The solid lines represent the RMS fitting of the data points to Equation 1, with shaded areas marking the fitting uncertainty. Error bars denote the standard deviation from 2-3 sample repetitions. The fitting curves from **Figure 2** are shown as dashed linen for comparison with the acidic impregnation experiments.

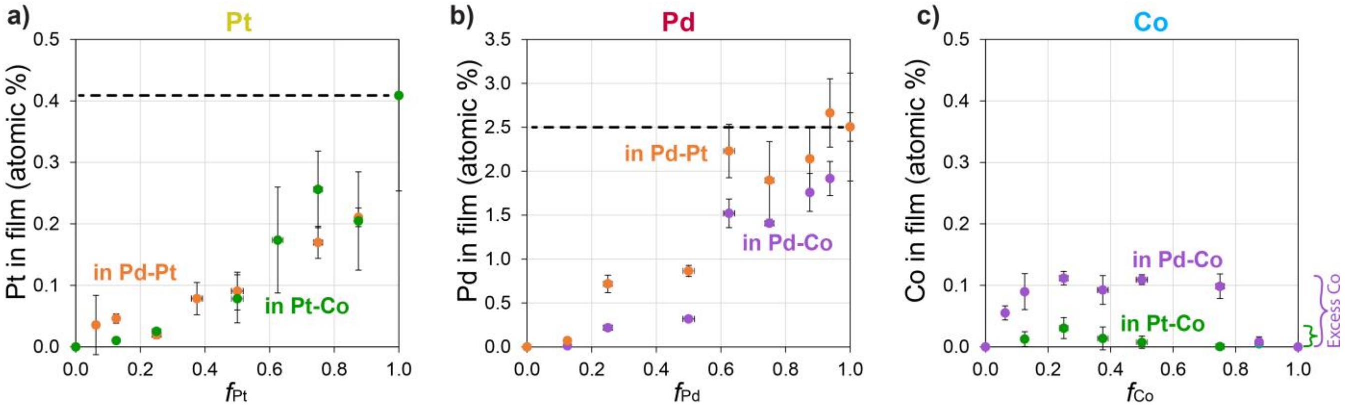

Figure S8. Atomic concentrations of a) Pt, b) Pd, and c) Co as measured by EDX, as a function of their molar fraction in the feed solution in the acid-free impregnation experiments. Each set of data points corresponds to the amount of one of the metals in a certain bimetallic pair. Error bars denote the standard deviation from 2-3 sample repetitions. The value at f =1 is marked with a dashed line; metal absorbed in excess above this value is denoted (applies to Co only in this case). Note the different vertical scales between the different metals.

Methodology for constructing the fitting equation

Fitting the datasets in **Figure 2** to the Mayo-Lewis copolymerization composition equation (Equation S1):

$$F_A = \frac{r_A f_A^2 + f_A f_B}{r_A f_A^2 + 2 f_A f_B + r_B f_B^2} \qquad \text{(S1)}$$

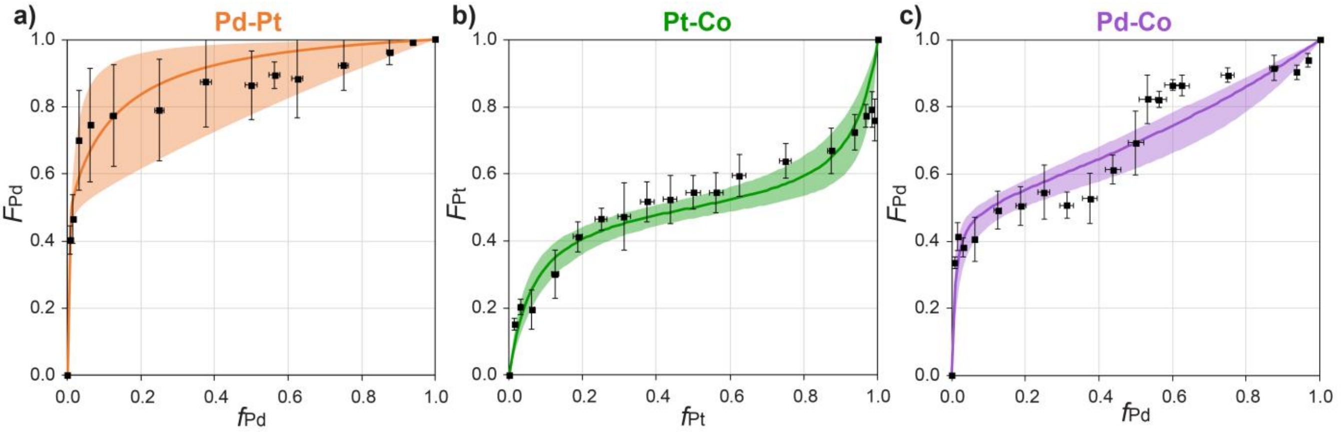

Figure S9. Fitting to Equation S1 for a) Pd-Pt, b) Pt-Co, c) Pd-Co, with shaded areas marking the fitting uncertainty. Error bars denote the standard deviation from 3-11 sample repetitions.

The fits for all metal pairs miss several data points, especially for Pd-Co, which boasts several rises in the curve (at f_{Pd} ≈ 0, 0.5, and 0.9). This suggested that an additional, slightly different expression has to be added to the equation (Equation S2):

$$F_A = \alpha \frac{r_A^+ f_A^2 + f_A f_B}{r_A^+ f_A^2 + 2 f_A f_B + r_B^+ f_B^2} + (1-\alpha) \frac{r_A^- f_A^2 - f_A f_B}{r_A^- f_A^2 - 2 f_A f_B + r_B^- f_B^2} \qquad \text{(S2)}$$

The two expressions differ only by the sign before the mixed term $f_A f_B$, denoting them as the "positive" and "negative" expressions. The corresponding parameters are denoted as r^+ and r^-, respectively.

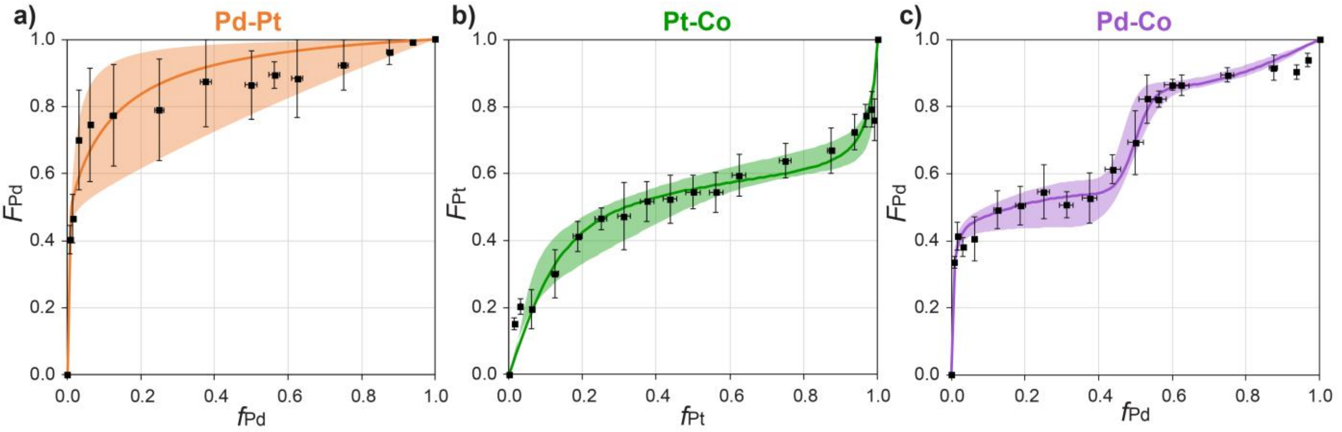

Figure S10. Fitting to Equation S2 for a) Pd-Pt, b) Pt-Co, c) Pd-Co, with shaded areas marking the fitting uncertainty. Error bars denote the standard deviation from 3-11 sample repetitions.

The fit for Pd-Pt (**Figure S10**a) remains exactly the same as in **Figure S9**a, meaning that the addition of the "negative" expression does not contribute to the fit (α=1). For Pd-Co and Pt-Co, however, it improves the fit considerably (**Figure S10**b,c). Yet, the fitting still seemed to miss the fine details of the trends, especially in the Pd-Pt and the high f_{Pd} data in the Pd-Co datasets. Thus the power of f_A and f_B was left as a free parameter P to find the optimal form of the equation (Equation S3):

$$F_A = \alpha \frac{r_A^+ f_A^{(2P)} + (f_A f_B)^{(P)}}{r_A^+ f_A^{(2P)} + 2(f_A f_B)^{(P)} + r_B^+ f_B^{(2P)}} + (1-\alpha) \frac{r_A^- f_A^{(2P)} - (f_A f_B)^{(P)}}{r_A^- f_A^{(2P)} - 2(f_A f_B)^{(P)} + r_B^- f_B^{(2P)}} \qquad \text{(S3)}$$

The best fit yielded P = 0.5 for all three pairs, giving the final Equation 1.

To better understand the contribution of each expression to the fit and its meaning to our system, we can look at the shape of the curves for each expression separately:

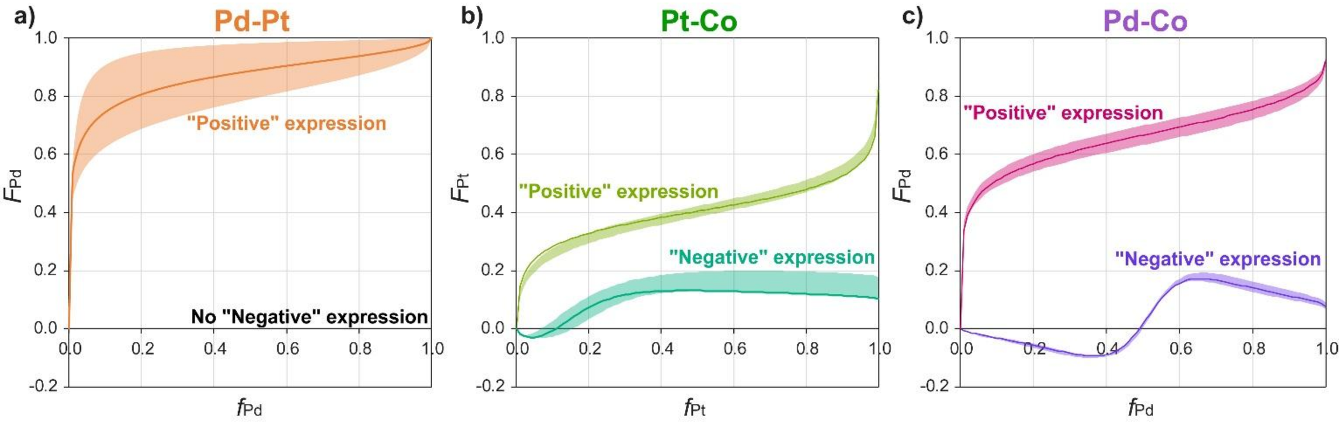

Figure S11. A breakdown of the fits for a) Pd-Pt, b) Pt-Co, and c) Pd-Co to the "positive" and "negative" expressions, according to Equation 1, with shaded areas marking the fitting uncertainty.

As mentioned in the text, the "positive" expression is associated with the competition mechanism. The curve for the "positive" expression of the Pd-Pt system (**Figure S11**a) resembles that of the common case in free radical copolymerization, where the binding to one of the metal precursors is highly favorable over the binding to the other (in analogy to the more reactive monomer in copolymerization, which features a reactivity ratio greater than 1, whereas the less reactive monomer feature a reactivity ration lower than 1). Indeed, r_{Pd}^{+}/r_{Pt}^{+} = 120, and $F_{Pd} \geq 0.5$ for almost all the feed range ($f_{Pd} > 0.008$).

The curves for the "positive" expression of the Pt-Co and Pd-Co (**Figure S11**b,c) feature sigmoidal shapes that resembles that of azeotropic copolymerization systems, which reflects a more "fair" competition (in the sense of only a slight preference of one metal precursor over the other and not a complete dominance). Indeed, the ratios r_{Pt}^{+}/r_{Co}^{+} = 0.68 and r_{Pd}^{+}/r_{Co}^{+} = 17.2 for the Pt-Co and Pd-Co systems, respectively, and the resulting composition is largely richer with the metal precursor that is in abundance. We also note here that the curves of the "positive" expression are shifted downward (Pt-Co) and upward (Pd-Co) from a sigmoidal curve that is symmetric about the F_{Pt} = 0.5 composition, which is consistent with the ratio of r^{+} values being either smaller (Pt-Co) or greater (Pd-Co) than unity. This results is also in accord with the preferred absorption of Pd over Co noted in the text, but it also suggests a slight preference of the absorption of Co over Pt. This means that the r^{+} values and their ratios could serve as a useful guide for predicting the relative affinities of metal precursor pairs to the pyridine functionalities in a P2VP film.

The curves for the "negative" expression, which appear only in the Pt-Co and Pd-Co systems, serve as modifiers to the "positive" expression curves. In the Pt-Co case (**Figure S11**b), the contribution of this expression is positive over most of the feed range, which is consistent with the cooperative effect that led to the introduction of excess Pt into the film (**Figure 3**a) in Pt-rich feeds.

In the Pd-Co case (**Figure S11**c), the contribution of the "negative" expression diverges from strongly negative at low f_{Pd} to strongly positive at high f_{Pd}. The latter is reflected by a large excess of impregnated Pd in this system at this feed range (**Figure 3**b), which is attributed to the cooperative effect as well. The negative portion of the "negative" expression (at $f_{Pd} < 0.48$) is somewhat surprising, and suggests that the $Co(CN)_6^{3-}$ actually hinders the binding of the Pd precursor (as reflected also in the **Figure 3**b, purple curve, which is lower at this range than the orange curve of the Pd-Pt pair). A plausible explanation for this behavior is that at this feed range the $Co(CN)_6^{3-}$ is the majority component, and because it is more negatively charged than the $PdCl_4^{2-}$ it impregnates the film faster. Then, incoming Pd precursors have to overcome not only the steric hindrance posed by the existing $Co(CN)_6^{3-}$ complexes but also the electrostatic barrier to be able to either displace these complexes or form a coordinative bond with the pyridine (in which case they will also have to break the N-H bond). At the higher f_{Pd} limit, this hindrance is diminished by the lower relative concentration of $Co(CN)_6^{3-}$ complexes and is also replaced by the ability to form the cyanide bridging, which is optimal at 2:1 Pd:Co ratios (f_{Pd} = 0.67).[5]

[1] H. B. Gray, N. Beach, *J. Am. Chem. Soc.* **1963**, *85*, 2922-2927.
[2] A. D. Torres, M. A. Francisco, R. R. Oliveira, A. B. Rocha, *J. Phys. Chem. A* **2023**, *127*, 3200-3209.
[3] L. I. Elding, L. F. Olsson, *J. Phys. Chem.* **1978**, *82*, 69-74.
[4] R. J. Deeth, *Faraday Discuss.* **2003**, *124*, 379-391.
[5] B. W. Pfennig, A. B. Bocarsly, R. K. Prud'homme, *J. Am. Chem. Soc.* **1993**, *115*, 2661-2665.
